# Regional Microarchitecture of the Skin of the Arabian Carpetshark (*Chiloscyllium arabicum*): A Histological Study Using Polarised Light Microscopy

**DOI:** 10.3390/ani16142118

**Published:** 2026-07-08

**Authors:** Anna Lipińska, Małgorzata Tarnowska, Konrad Cyprych, Małgorzata Bednarska, Jakub Kordas, Maciej Janeczek, Piotr Kuropka

**Affiliations:** 1 Department of Biostructure and Animal Physiology, Faculty of Veterinary Medicine, Wrocław University of Environmental and Life Sciences, Norwida 31, 50-375 Wrocław, Poland; malgorzata.tarnowska@upwr.edu.pl (M.T.); maciej.janeczek@upwr.edu.pl (M.J.); piotr.kuropka@upwr.edu.pl (P.K.); 2 Soft Matter Optics Group, Wrocław University of Science and Technology, Wybrzeże Wyspiańskiego 27, 50-370 Wrocław, Poland; konrad.cyprych@pwr.edu.pl; 3 Department of Epizootiology with the Clinic of Birds and Exotic Animals, Wrocław University of Environmental and Life Sciences, Norwida 25, 50-635 Wrocław, Poland; malgorzata.bednarska@upwr.edu.pl; 4 Wrocław Zoo, 1–5 Wróblewskiego Street, 51-618 Wrocław, Poland; j.kordas@zoo.wroc.pl

**Keywords:** elasmobranch, dermal denticles, collagen organisation, anisotropy, polarised light microscopy, shark skin, functional morphology

## Abstract

The integument of elasmobranchs is a complex biomechanical system in which epithelial, connective tissue and mineralised components form a functionally specialised body surface. Despite extensive research on dermal denticles, the spatial configuration of the whole integument, especially the regional arrangement of collagen fibres in benthic species, remains poorly understood. This study provides an integrated characterisation of the skin microarchitecture of the Arabian carpetshark (*Chiloscyllium arabicum*), with emphasis on regional variability in dermal denticles and collagen matrix structuring. Skin samples were analysed using haematoxylin and eosin, Masson–Goldner, PAS–Alcian blue and picrosirius red staining, together with transmitted and polarised light microscopy and morphometric analysis. The skin of this species showed a hierarchical organisation comprising a stratified epithelium and a complex fibrous dermis. Dermal denticles displayed typical elasmobranch anatomy, but their morphology, orientation, and density varied regionally, allowing five morphotypes to be distinguished. Collagen fibres formed ordered, plywood-like and orthogonal arrangements showing spatial correspondence with local denticle distribution. These findings indicate regional structural specialisation of the integument and suggest a structural association, and a possible functional relationship, between denticle morphology and collagen architecture.

## Simple Summary

Shark skin is more than a protective covering; it is a specialised surface that helps the animal interact with its environment. In bottom-dwelling sharks, this surface may be particularly important because it is exposed to both water flow and repeated contact with the seabed. This study examined the skin of the Arabian carpetshark (*Chiloscyllium arabicum*) to determine how its surface structures and deeper connective tissue are organised across different body regions. Microscopic analysis showed a layered structure consisting of an outer cell layer, small tooth-like dermal denticles, and a deeper layer rich in collagen fibres. Five main denticle forms were identified, and their distribution varied across the body. The arrangement of collagen fibres also differed regionally, ranging from ordered patterns to more complex interwoven networks. These findings indicate that the skin of this species is regionally specialised rather than uniform. The study provides new information on shark skin organisation and may help explain how aquatic animals adapt their body surfaces to mechanical and environmental demands.

## 1. Introduction

The integument of sharks represents a complex, multi-layered morphological system shaped by ecological, biomechanical, and evolutionary adaptations. In Chondrichthyes, the skin forms a functionally integrated unit composed of the epidermis, dermis, and dermal denticles [1,2,3,4,5,6]. Together, these components contribute to mechanical load distribution [3,7,8,9,10], modulation of near-body water flow and hydrodynamic performance [4,11,12,13,14,15,16,17], and protection [1,5,6]. Shark skin is, therefore, not only a protective covering but also a biomechanical interface involved in locomotion and mechanical loading [3,10,11,12,13,14,15,16,17,18,19], while dermal-denticle topography contributes to interactions with near-body flow and the external environment [4,5,6,11,12,13,14,15,16,17].

Among Elasmobranchii, collagen fibre organisation and dermal mechanical properties vary with species, ecomorphology, and body region [3,7,8,9,10], whereas dermal-denticle morphology, surface roughness, and ornamentation vary substantially among taxa and body regions. Dermal denticles form a continuum of morphologies, from smooth, flat structures to ornamented crowns with surface ribs [1,5,6,11,20,21,22]. In pelagic and highly mobile species, such as shortfin mako shark *Isurus oxyrinchus* and the blacktip shark *Carcharhinus limbatus*, slender crowns with parallel ridges may reduce near-wall turbulence and hydrodynamic drag [4,11,12,13,14,15,16,17]. Recent three-dimensional, computational and comparative studies further indicate that denticle morphology, ridge geometry, surface roughness, and mechanical performance vary substantially among shark species and body regions, supporting the interpretation that shark skin functions as a heterogeneous rather than uniform biological surface [5,6,12,14,15,16,17,20]. In contrast, reduced and robust denticle forms in benthic sharks, including Hemiscylliidae, should be presented as a plausible adaptation to abrasion and local mechanical loading, supported primarily by comparative denticle-function literature [5,6,11,22], whereas distributional and reproductive sources on *C. arabicum* support species context rather than direct abrasion mechanics [23,24].

The dermis is an important structural component of the shark integument and is characterised by a hierarchical organisation of type I collagen fibres. These fibres are arranged in alternating layers, forming a cross-ply architecture directly supported by shark-skin and fish-skin mechanical studies [3,7,8,9,10,18,19], with broader biomechanical context provided by [25]. Collagen bundles, typically oriented obliquely to the longitudinal body axis, confer anisotropic properties on the dermis and enable it to withstand complex mechanical stresses, including tension, friction, and torsion [3,10,18,19]. Recent mechanical analyses also show that shark skin properties vary with body region, sex, species, ecomorphology, and ontogenetic stage, indicating that collagen architecture and denticle morphology jointly contribute to regional mechanical behaviour [5,7,8,9,10]. In parallel, the epidermis contains specialised mucus-secreting goblet cells, which produce mucins involved in surface protection and friction reduction [26], while pigment cells may contribute to body-surface colouration and camouflage [2].

Comparative studies indicate that shark dermis and denticle morphology may vary substantially among ecomorphological groups and body regions, which may reflect adaptation to different mechanical and environmental demands [5,6,7,8,9,10]. Despite growing knowledge of shark skin morphology, detailed analyses of regional integument structure in benthic species remain limited. In particular, few studies have integrated collagen architecture, epidermal cell distribution and dermal denticle microarchitecture using complementary histochemical and microscopic techniques.

Considering these gaps, this research focuses on the arabian carpetshark, *Chiloscyllium arabicum*, a benthic species occurring in the Arabian/Persian Gulf and Northern Arabian Sea region, currently according to the IUCN Red List classified as Near Threatened (NT) [23,24,27]. Consequently, the aim of this study was to perform integrated analysis of the regional microarchitecture of the integument of *C. arabicum*, with particular emphasis on collagen fibre organisation, dermal denticle morphology, and epidermal cell arrangement.

## 2. Materials and Methods

### 2.1. Animals

Skin samples from one adult female *C. arabicum*, obtained post-mortem from Wrocław Zoo in accordance with institutional procedures for the use of post-mortem material, were used in this research. The individual was 64 cm long and had reached sexual maturity. Before death, the individual showed no macroscopic changes in the integument or any signs of skin disease.

### 2.2. Ethical Statement

The research material was obtained post-mortem and transferred to the Faculty of Veterinary Medicine, Wrocław University of Environmental and Life Sciences, in accordance with applicable Polish law (Act of 15 January 2015 on the protection of animals used for scientific or educational purposes, Journal of Laws of the Republic of Poland 2015, item 266) and the procedures of the unit providing the material. The study was conducted exclusively on post-mortem material obtained from an animal whose death was unrelated to the purpose of the present investigation or to any skin pathology and resulted from the presence of a foreign body in the stomach. No procedures were performed on live animals for the purpose of this study, including handling, anaesthesia, euthanasia, or any other interventions. Under Polish and European Union legislation, studies using only post-mortem material and involving no procedures on live animals do not require approval from the competent ethics committee. Therefore, ethical review and approval were not required.

### 2.3. Histological Analysis

Single skin sections from topographically distinct body regions were included in the analysis (*n* = 1 per region). Sample selection was oriented toward assessing the regional variability of the architectural and histological organisation of the integument; no population-level quantitative analyses were conducted. As this research was based on a single individual, the results should be interpreted as a detailed morphological study rather than a population-level analysis.

**Figure 1:**
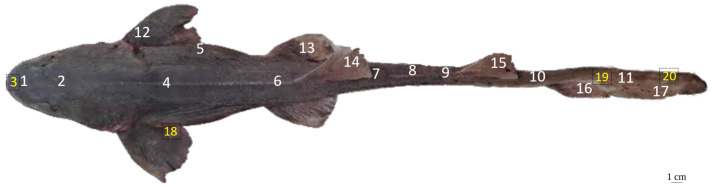
Skin sampling sites in *Chiloscyllium arabicum* (20 body regions) included dorsal regions (1, 2, 4, 6–10), a lateral region (5), fin-associated regions (11–15), ventral regions (3, 18–20), and ventrolateral/caudal ventral regions (16, 17). Regions marked in yellow squares indicate sampling points located on the ventral surface. Scale bar: 1 cm.

Skin sections measuring approximately 0.5 × 0.5 cm were collected from 20 defined topographical body regions of *C. arabicum* (Figure 1). The sampling sites were selected to represent distinct dorsal, lateral, ventral, fin-associated, and transitional regions, enabling comparison of integument organisation along the dorsoventral and anteroposterior axes and accounting for differences in mechanical exposure and substrate contact. These included dorsal regions (1, 2, 4, 6–10), the lateral region (5), fin-associated regions (11–15), ventral regions (3, 18–20), and ventrolateral and caudoventral regions (16, 17). After collection, the skin samples were processed according to a standard histological workflow.

All samples were fixed in 4% buffered formaldehyde (pH 7.2–7.4) for five days and then rinsed in running water for 12 h according to standard histological procedures [28]. Due to the presence of mineralised dermal denticles, the material was decalcified to enable microtome sectioning [29]. After decalcification, the samples were rinsed again in water, dehydrated through an ascending ethanol series, cleared in xylene, infiltrated with paraffin, and embedded in paraffin blocks [28]. During embedding, the samples were oriented to obtain sections perpendicular to the skin surface, allowing assessment of the epidermis, dermis, and dermal denticles in the same plane. Paraffin blocks were sectioned at 5 µm using a rotary microtome, and the sections were mounted on glass slides.

Sections were stained with haematoxylin and eosin (H&E) to assess the general organisation of the epidermis, dermis, and dermal denticles; Masson–Goldner trichrome to visualise connective tissue; and the PAS–Alcian blue method to identify carbohydrate components and differentiate between acidic and neutral mucins [28,30]. All reagents were purchased from Merck KGaA (Merck KGaA, Darmstadt, Germany). Additional sections were stained with Picrosirius Red (Polysciences, Inc., Warrington, PA, USA) to evaluate collagen fibre organisation under polarised light. For this staining, deparaffinised and rehydrated sections were incubated in 0.1% Sirius Red F3B dissolved in saturated aqueous picric acid for 60 min, rinsed in acidified water, rapidly dehydrated in ethanol, cleared in xylene, and mounted with a resinous medium. Picrosirius Red staining was used to enhance collagen birefringence and assess the arrangement and distribution of collagen fibres under polarised light, following established histochemical protocols [31,32,33].

Epidermal thickness was measured exclusively in well-preserved sections, excluding areas that were mechanically distorted or affected by sectioning artefacts. The obtained values were treated as relative measures for regional comparisons rather than absolute in vivo values.

Histological evaluation of stained sections was performed using a Axio Scope A1 (Carl Zeiss, Jena, Germany) coupled to AxioVision, AxioVs40, V 4.8.2.0 image analysis software. This system was used to assess the microscopic organisation of the epidermis, dermis, and dermal denticles and to acquire images for morphometric measurements. 

Macroscopic assessment of whole skin sections and dermal denticle distribution was performed using a Stemi 508 stereomicroscope equipped with an Axiocam 208 colour camera (Carl Zeiss, Jena, Germany) and ZEISS ZEN 3.1 (blue edition) software.

Morphometric analysis of the dermal denticles and epidermal thickness was performed using Fiji 2.17.0 software (ImageJ 2.16.0; Java 1.8.0_452).

Morphometric analysis was descriptive and was performed by a single trained observer. For each body region, 5–10 complete and clearly visible dermal denticles were assessed within the analysed field, depending on tissue preservation and the availability of structures suitable for reliable measurement. Denticles that were damaged, folded, partially sectioned, poorly oriented, or out of focus were excluded. All measurements were performed on calibrated digital images and repeated twice; discrepant values were re-evaluated using the original micrograph. Because the study was based on a single individual and one section per region, no inferential statistical analyses were performed, and the morphometric data are presented as descriptive regional measurements.

### 2.4. Analysis of Collagen Fibre Organisation Under Polarised Light

Skin samples stained with picrosirius red were observed under polarised light using a Delta Optical microscope. Images were recorded using a DLT-Cam PRO 6.3 MP USB 3.0 camera. The arrangement and orientation of type I and III collagen fibres were assessed on the basis of birefringence patterns and interference colours, in accordance with the criteria described previously [31,32,33], using Delta Optical DLT-Cam Viewer 20230808 software. Thick, densely packed collagen fibres showing strong birefringence and yellow-orange to red interference colours were interpreted as being consistent with predominantly type I collagen, whereas thinner, more delicate fibres showing weaker birefringence and greenish interference colours were interpreted as being consistent with predominantly type III collagen; however, this interpretation should be treated as histochemical and optical inference rather than definitive collagen typing. Fibre arrangement was assessed according to the degree of parallel alignment, waviness, crossing, and dispersion of collagen bundles within the dermis.

Regional variations in collagen fibre orientation were further analysed using two-dimensional Fast Fourier Transform (2D FFT) analysis of selected polarised light microscopy images. Exposure times ranged from 4.27 to 8.27 ms. Image processing and FFT analysis were performed in Fiji/ImageJ software using the built-in FFT tools [34]. Before analysis, only well-preserved image areas were selected, excluding regions with folds, tears, compression artefacts, uneven staining, out-of-focus areas, air bubbles, or tissue discontinuities. Regions of interest (ROIs) were selected manually on the basis of clearly visible collagen fibre patterns and preserved tissue architecture.

Colour-coded maps were generated by synthesising a series of micrographs captured using crossed polarisers at various sample orientations relative to the polarisation axis; this approach allowed the reconstruction of the complete birefringence signal of the collagen fibres. For FFT analysis, the combined polarised light images were converted to 8-bit grayscale and, where necessary, adjusted for brightness and contrast without altering the spatial relationships of the structures. No automated segmentation or threshold-based fibre extraction was applied. A 2D FFT was then performed on the selected ROIs to identify dominant periodic and directional components within the collagen fibre network.

Fibre orientation was determined based on local image intensity gradients, enabling the identification of specific directional patterns within the analysed skin regions. For orientation analysis, fibres were classified as predominantly parallel when they showed a common dominant direction, multidirectional when several fibre orientations were present within the same field, and irregular when no consistent directional pattern could be identified. Only well-preserved areas without folding, tearing, compression, or staining artefacts were selected for orientation analysis. In the FFT output, the position of high-intensity bands or peaks was interpreted as indicating the dominant spatial periodicity and angular distribution of collagen structures within the analysed ROI. Periodic fibre organisation was recognised when repeated bands or discrete intensity peaks were visible in the FFT spectrum and corresponded to regular collagen patterns observed in the original microscopy image. Fibre orientation and periodicity were therefore assessed semi-quantitatively: the FFT analysis was used to support visual interpretation of collagen organisation rather than to provide fully automated fibre tracking. All ROIs and FFT outputs were inspected manually to verify correspondence between the original image and the frequency-domain pattern.

The source materials, including paraffin blocks and histological sections, were deposited in the reference collection of the Laboratory of Histology and Embryology at the Department of Biostructure and Animal Physiology, Wrocław University of Environmental and Life Sciences.

## 3. Results

### 3.1. Dermal Denticle Morphotypes and Morphometrics

**Figure 2:**
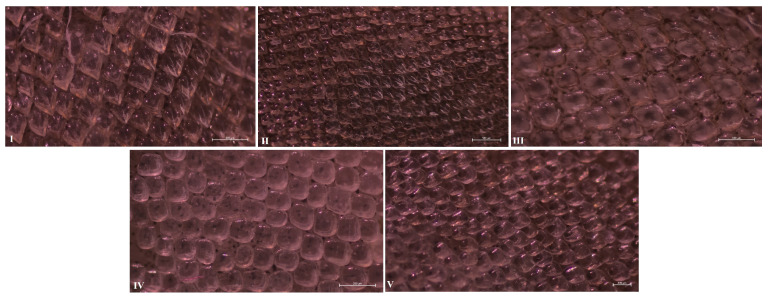
Skin surface of *Chiloscyllium arabicum* with dermal denticles. Five distinct morphological types of dermal denticles are shown; panel numbers correspond to morphotype numbers: (**I**)—region 8, (**II**)—region 5, (**III**)—region 12, (**IV**)—region 20, (**V**)—region 17. Scale bars: 500 µm (panels **I**–**IV**); 200 µm (panel **V**).

*C. arabicum* exhibited a pronounced regional gradient in dermal denticle morphology along the dorsoventral axis (Figure 2, Table 1). Morphometric assessment included 5–10 complete and clearly visible dermal denticles per field of view. The mean denticle surface area was 1.3 × 10^5^ µm^2^. Based on crown geometry, surface ornamentation, and the degree of structural differentiation, five primary morphotypes were identified.

Morphotype I: Elongated ridge-bearing type. This morphotype occurred in the dorsal regions (1, 2, 4, and 6–10) and was characterised by an elongated, anisotropic crown with a distinct posteriorly directed cusp. Its diagnostic features included a well-developed system of longitudinal ridges and clear morphological separation between the crown, neck, and base (Figure 2, Table 1).

Morphotype II: Flattened tile-like type. This morphotype was specific to the lateral region (5). It comprised denticles with a low-profile, isotropic crown lacking a distinct cusp. Surface ornamentation was strongly reduced, and the neck was indistinct or vestigial, resulting in a smooth transition from the crown to a broad base. Denticles of this type formed a regular, closely packed tessellated pattern (Figure 2, Table 1).

Morphotype III: Dome-shaped fin type. This morphotype occurred in the fin regions (11 and 13–15). It was characterised by a low to moderately dome-shaped crown with a rounded, rounded-quadrangular, or sub-square outline. The crown surface was smooth or only subtly sculptured, lacking both a developed cusp and distinct ridges. In contrast to morphotype IV, the crown remained morphologically distinguishable despite limited differentiation of the neck and base. This morphotype showed moderate internal variation: region 11 was dominated by more isotropic forms, whereas regions 13–15 more frequently contained sub-square crowns arranged in regular rows parallel to the longitudinal body axis (Figure 2, Table 1).

Morphotype IV: Reduced plate-like type. This morphotype occurred on the ventral surface (3 and 18–20) and represented the most reduced form of dermal denticles observed in this study. It was characterised by a very low, dome-shaped to plate-like crown, devoid of surface ornamentation, cusps, or ridges. Its diagnostic feature was marked structural reduction, expressed by fused crown–base morphology and the absence of a distinct neck (Figure 2, Table 1).

Morphotype V: Transitional reduced type. This morphotype was identified in regions 16 and 17 and represented an intermediate form between denticles with more developed crowns and highly reduced types. It was characterised by a small, low-profile, and often asymmetrical crown with an irregular outline, smooth surface, and poorly defined crown–neck–base organisation. Its distinctiveness resulted from the consistent co-occurrence of irregular crown shapes, limited structural differentiation, and the absence of an organised spatial arrangement of the denticles (Figure 2, Table 1).

The differentiation of denticle morphotypes was accompanied by regional differences in the inclination angle of the crowns relative to the integumentary surface. Mean orientation values ranged from 22.33° to 53.09° among the analysed regions. Denticles assigned to morphotype I showed the highest degree of anisotropy and the most directional crown orientation, whereas reduced forms assigned to morphotypes IV and V were characterised by more isotropic, low-profile, and flat-set arrangements.

### 3.2. General Histological Structure of the Integument

**Figure 3:**
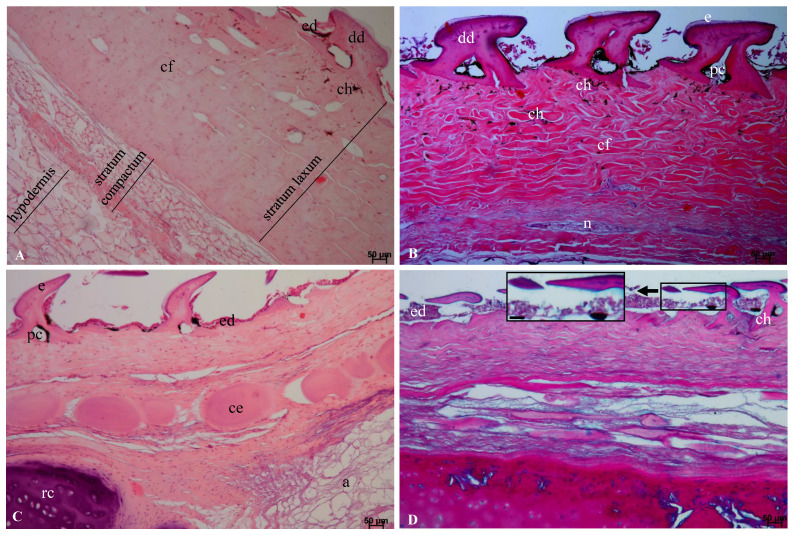
Histological organisation of the integument in *Chiloscyllium arabicum*. Haematoxylin and eosin (**A**,**C**) and PAS–Alcian blue (**B**,**D**) staining. Panels show representative histological features from selected body regions: (**A**)–region 7, (**B**)–region 1, (**C**)–region 16, (**D**)–region 17; dd, dermal denticles; e, enameloid; ed, epidermis; cf, collagen fibres; ce, ceratotrichia; rc, radial cartilage; a, adipocytes; ch, chromatophore; n, nerves; pc, pulp cavity. Scale bars: 50 µm (**A**–**C**) and 20 µm (**D**).

**Figure 4:**
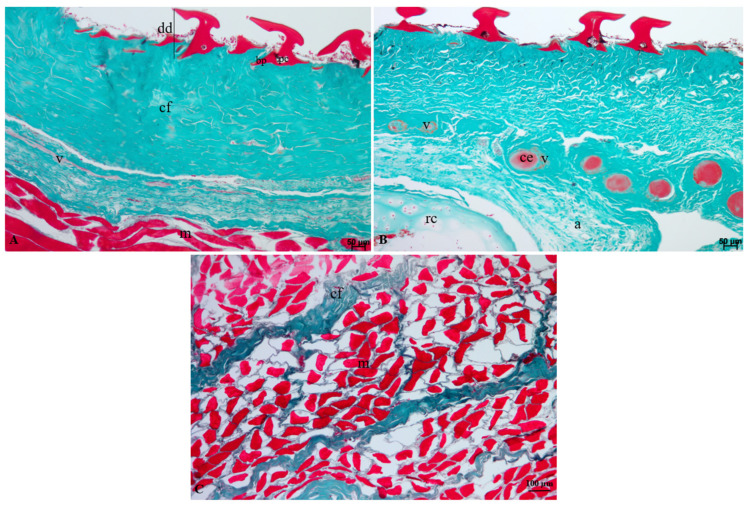
Histological organisation of the integument in *Chiloscyllium arabicum*. Masson–Goldner trichrome staining (**A**–**C**). Panels show representative dermal and subcutaneous features from selected body regions: (**A**)–region 1, (**B**)–region 12, (**C**)–region 5; dd, dermal denticles; cf, collagen fibres; ce, ceratotrichia; rc, radial cartilage; a, adipocyte; m, muscle; bp, basal plate; pc, pulp cavity; v–vein. Scale bars: 50 µm (**A**,**B**) and 100 µm (**C**).

The skin of *C. arabicum* exhibited a distinctly organised, stratified architecture comprising a superficial epidermis and an underlying dermis containing densely distributed dermal denticles (Figure 3). The general histological organisation described in this section was assessed across the analysed regions, whereas Figure 3 and Figure 4 show representative examples from selected body regions. Histologically, the integument formed a spatially integrated structural complex, in which the covering epithelium was supported by an extensive fibrous connective tissue stroma containing mineralised elements. The crowns of the dermal denticles protruded above the integumentary surface, creating a micro-ornamented surface topography characterised by a regular, although locally heterogeneous, distribution across the body.

The integumentary structure was organised into three primary morphological levels: a superficial stratified epidermis, mineralised dermal denticles with regionally variable morphology, and a complex fibrous dermal stroma. Chromatophores showed a multilayered distribution (Figure 3B). Melanophores were most abundant in the subepithelial layer immediately beneath the epidermis, corresponding to the superficial dermis. Additional pigment clusters were localised in the deeper dermis, particularly near the denticle bases and within the central pulp cavity of the dermal denticles (Figure 3A,B,D).

#### 3.2.1. Epidermis

The epidermis consisted of a stratified epithelium measuring 20–27 µm in thickness and typically comprising three to four cell layers. This organisation was observed across the analysed regions, with no marked regional differences in the basic architecture of the epidermis. It continuously covered the interspaces between the crowns and necks of the dermal denticles (Figure 3D). The predominant cell population consisted of flattened epithelial cells with intensely basophilic nuclei and eosinophilic cytoplasm. The dermoepidermal junction followed an undulating course, reflecting the underlying topography of the denticle bases.

Specialised mucous cells were clearly distinguishable within the epidermis. PAS–Alcian blue staining revealed a strong positive cytoplasmic reaction, confirming the presence of acidic and neutral polysaccharides (Figure 3D). These cells had pale, vacuolated cytoplasm and were associated with the mucus-producing component of the epidermis. Pigment cells were also observed sporadically near the basal layer, particularly overlying the denticle bases.

#### 3.2.2. Dermis and Subcutaneous Layer

The dermis exhibited a highly ordered, bilayered organisation, clearly distinguishable in haematoxylin and eosin (H&E) and Masson–Goldner trichrome staining (Figure 3A and Figure 4B). This bilayered organisation was present in the examined regions; however, local differences were observed in the relative development of the fibrous dermal layers, the embedding of dermal denticles, and the presence of fin-associated or hypodermal structures. The superficial portion of the dermis corresponded to the stratum laxum, which formed a dense fibrous zone composed of tightly packed collagen bundles oriented predominantly parallel to the body surface. This layer surrounded the denticle bases and contributed to their embedding within the integument.

The deeper portion corresponded to the stratum compactum, which showed a more complex spatial organisation. This layer contained blood vessels, nerves, and small bundles of muscle fibres interspersed with connective tissue elements (Figure 4C). In the fin regions, ceratotrichia were visible as elongated fibrous support structures arranged in a single row. The integument was underlain by the hypodermis, which contained a distinct and continuous adipocyte layer separating the dermis from the underlying musculature (Figure 3C and Figure 4B).

### 3.3. Organisation of Collagen Fibres

**Figure 5:**
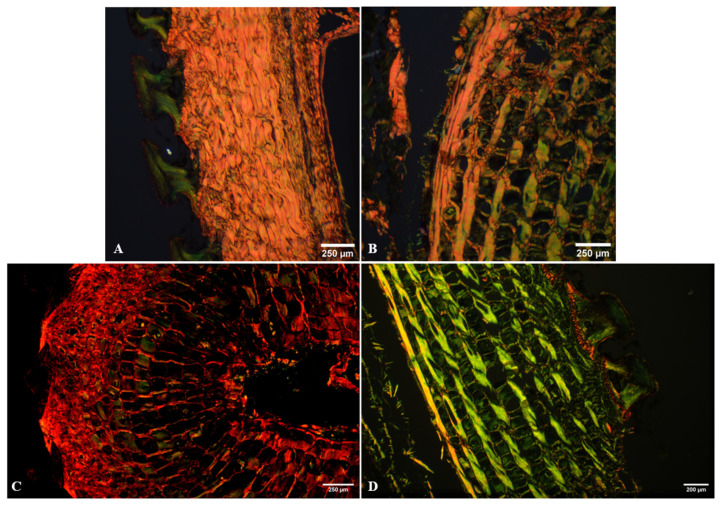
Regional variation in collagen fibre orientation in the integument of *Chiloscyllium arabicum* under polarised light. (**A**) Periodic birefringence patterns (region 1); (**B**) predominantly aligned collagen fibres (region 7); (**C**) thick and thin collagen bundles (region 4); (**D**) collagen beneath dermal denticles (region 7). Scale bars: 250 µm (**A**–**C**) and 200 µm (**D**).

**Figure 6:**
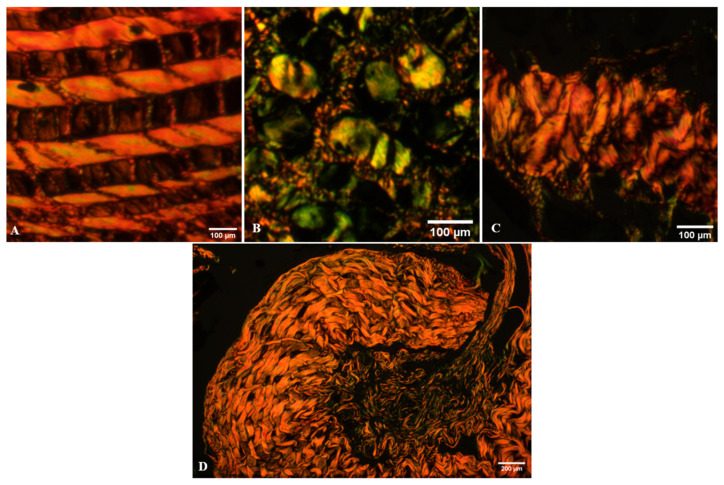
Interwoven collagen fibre networks in the integument of *Chiloscyllium arabicum* under polarised light. (**A**) Oblique interweaving of collagen bundles (region 6); (**B**) twisted collagen bundles (region 7); (**C**) dense collagen fibre network (region 7); (**D**) heterogeneous collagen organisation (region 18). Scale bars: 100 µm (**A**–**C**) and 200 µm (**D**).

**Figure 7:**
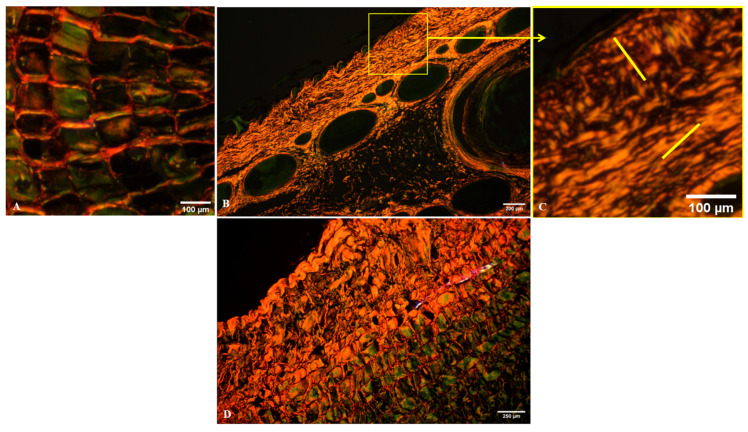
Collagen organisation associated with adjacent tissue structures in the integument of *Chiloscyllium arabicum*. (**A**) Collagen fibres surrounding adipocytes (region 4); (**B**) collagen fibres surrounding fin cartilage (region 12); (**C**) collagen bundles oriented perpendicular and parallel to the surface (region 12); (**D**) layered collagen organisation (region 9). The area outlined by the yellow square shows a magnified view of the organization of collagen bundles within the fin region (region 12). Scale bars: 100 µm (**A**,**C**), 200 µm (**B**), and 250 µm (**D**).

Polarised light microscopy revealed distinct regional differentiation in the organisation of collagen fibres within the skin of *C. arabicum*, ranging from uniformly oriented structures to periodic and interwoven arrangements (Figure 5, Figure 6 and Figure 7; Table 2). This analysis was performed comparatively across selected representative regions, and regional differences were interpreted in relation to the topographical origin of the samples and the local organisation of the dermis.

In selected regions, particularly regions 7 and 11, a homogeneous fibre orientation predominated, indicating a strongly directional organisation of the collagen matrix (Figure 5B; Table 2). In areas located directly beneath the dermal denticles, ordered but looser fibrous arrangements were observed, with bundles interwoven at defined angles (Figure 5D).

In other regions, including regions 1 and 4, periodic fibre organisation was identified and was associated with regular changes in the polarisation signal (Figure 5A,C; Table 2). Two-dimensional Fast Fourier Transform (2D FFT) analysis confirmed the presence of repetitive structural patterns with characteristic periodicity. The FFT spectra showed distinct high-intensity bands or peaks corresponding to the periodic and directionally organised collagen patterns observed in the original polarised light microscopy images.

Several regions, including regions 6, 7, and 18, exhibited complex networks of interwoven fibres of varying thicknesses, forming lattice-like arrangements (Figure 6A–D; Table 2). In some areas, adipocytes were embedded within this fibrous matrix. Fibres were arranged concentrically around fat cells (Figure 7A), whereas orthogonal and undulating fibre arrangements were observed near cartilaginous elements (Figure 7B,C).

In region 9, a distinct layered organisation was identified, corresponding to a plywood-like, cross-lamellar arrangement characteristic of elasmobranch skin (Figure 7D; Table 2). Overall, collagen fibre organisation showed continuous spatial variability, ranging from uniformly oriented systems through periodic arrangements to multiscale interwoven networks (Table 2).

#### 3.3.1. Regions Exhibiting Periodic Fibre Organisation

In several regions, periodic changes in the polarisation signal were observed, corresponding to regular twisting or undulation of collagen bundles (Figure 5A,C; Table 2). In region 1, the mean fibre orientation was 73.5° ± 18.15°. The central portion of this area was characterised by lower fibre density, with a distance between interference fringes of 6–8 µm (Figure 5A).

Image analysis using 2D FFT revealed a dominant structural periodicity of approximately 140 µm, corresponding to a repetitive organisational pattern of collagen fibres (Figure 5A; Table 2). A similar organisation was observed in region 4, where two fibre populations were present: thicker bundles measuring approximately 75 µm and thinner fibres measuring approximately 15 µm. The corresponding polarisation pitches were approximately 100 µm and 12 µm, respectively (Figure 5C).

In subregion 7, thick collagen bundles measuring approximately 80 µm were interwoven with thinner fibres measuring approximately 15 µm, with a polarisation pitch of approximately 45 µm (Figure 6B,C; Table 2).

#### 3.3.2. Regions with Interwoven Fibre Organisation

In several areas, collagen formed complex networks of interwoven fibres with variable diameters (Figure 6A–D; Table 2). In region 6, thick collagen bundles were aligned in a dominant direction and interwoven with thinner fibres measuring approximately 15 µm at an angle of approximately 40° (Figure 6A). The presence of adipocytes within the fibrous matrix created distinct interstitial spaces of approximately 90 µm (Figure 7A).

A similar organisational type was observed in subregion 7 and region 18, where collagen formed dense networks of interwoven fibres across a wide size range, from approximately 7 to 100 µm (Figure 6B–D; Table 2).

#### 3.3.3. Collagen Organisation Associated with Cells and Cartilage

In region 4, adipocytes measuring approximately 110 × 130 µm were observed. The surrounding collagen fibres formed concentric or semi-concentric arrangements around the fat cells (Figure 7A).

In region 12, cartilaginous elements of the fins were present. Collagen fibres surrounding these structures formed highly corrugated patterns with an orthogonal organisation, with vertically oriented fibres in the outer layer and horizontally oriented fibres in the inner layer. Each layer measured approximately 200 µm in thickness (Figure 7B,C).

#### 3.3.4. Layered Organisation of the Skin

A distinct layered organisation of collagen was observed in region 9. The sub-denticular layer, located directly beneath the dermal denticles, reached a thickness of approximately 950 µm, whereas the deeper layer measured approximately 1200 µm (Figure 7D). Birefringence patterns indicated a multilayered arrangement of collagen fibres corresponding to a plywood-like architecture (Figure 7D; Table 2).

### 3.4. Regional Co-Occurrence of Dermal Denticle Morphotypes and Collagen Organisation

In the examined skin regions, specific dermal denticle morphotypes co-occurred with distinct patterns of collagen fibre organisation within the dermis (Table 1 and Table 2). Morphotype I, present in regions 1, 2, 4, and 6–10, was associated with diverse collagen arrangements, including periodic and interwoven systems (Figure 5A,C,D and Figure 6A–C; Table 1 and Table 2). Undulating structures, entangled networks, and fibres of variable diameters were also observed in these areas (Figure 6A–C; Table 2).

In regions 11–15, corresponding to morphotype III, the dermis exhibited a more homogeneous or locally ordered organisation, with a predominance of a single fibre orientation (Figure 5B; Table 1 and Table 2). Looser, oriented fibrous systems were also visible directly beneath the denticles (Figure 5D).

Morphotype IV, occurring in regions 3 and 18–20, co-occurred with a multiscale interwoven collagen network composed of densely packed fibres with a wide range of diameters (Figure 6D; Table 1 and Table 2). In some areas, this organisation was associated with the presence of adipocytes (Figure 7A; Table 2).

Morphotype V, identified in regions 16 and 17, corresponded to an interwoven and heterogeneous collagen arrangement characterised by high local variability and the absence of a clearly dominant fibre direction (Table 1 and Table 2). In region 5, assigned to morphotype II, no data were obtained that allowed a definitive assessment of collagen organisation (Table 1).

## 4. Discussion

The present study demonstrates pronounced regionalisation of the integument in *C. arabicum*, expressed through coordinated variation in dermal denticle morphology and collagen fibre architecture. This heterogeneity does not appear to represent random structural variation but rather a spatially organised pattern, suggesting that the skin functions as an integrated composite system in which mineralised denticles and fibrous dermal components jointly contribute to local mechanical performance [3,5,6,7,8,9,10]. This interpretation is consistent with the established view of shark skin as a composite tissue composed of a multilayered epidermis, a fibrous dermis and mineralised dermal denticles [1,2,3,4,5,6]. Because *C. arabicum* is a benthic representative of the order Orectolobiformes, the present findings should be interpreted primarily in relation to closely related carpet sharks and other bottom-associated elasmobranchs rather than solely through comparisons with fast-swimming pelagic species. However, detailed comparative data on regional relationships between dermal denticle morphology and collagen fibre organisation remain limited for benthic Orectolobiformes. Biochemical data are available for skin collagen from a related *Chiloscyllium* species [35], but these data do not resolve regional dermal architecture.

From an ecological perspective, substrate contact, local abrasion, body flexion, compression, and multidirectional loading are likely to be particularly relevant to the organisation of the integument in benthic sharks. Comparisons with related or ecologically similar taxa should therefore distinguish between phylogenetic similarity and functional convergence associated with a bottom-dwelling lifestyle.

Accordingly, all functional interpretations in the following discussion should be regarded as hypotheses derived from the morphology of a single adult female rather than as demonstrated species-level, sex-independent, or ontogenetically stable patterns.

The general organisation of the dermis into a superficial stratum laxum and a deeper stratum compactum corresponds to the conservative elasmobranch pattern [1,2,3,4,9]. In *C. arabicum*, however, this arrangement shows clear regional differentiation. Dorsal regions, corresponding to morphotype I, exhibited elongated, anisotropic denticles accompanied by more ordered collagen orientation, including periodic and interwoven systems. Such configurations may contribute to directional stiffness and force transmission along the body axis, consistent with the biomechanical role of collagenous fish and shark skin reported previously [3,10,18,19] and with recent studies showing that shark skin mechanics vary with body region, stress direction, and ecomorphological context [7,8,9,10]. Although ridge-bearing denticles are commonly discussed in relation to hydrodynamic performance in pelagic sharks [36,37], their occurrence in the dorsal regions of a benthic carpet shark should not be interpreted exclusively as a drag-reducing specialisation. In the examined specimen of *C. arabicum*, these structures may also be associated with local surface protection, resistance to deformation, and mechanical reinforcement in regions exposed to body flexion or external contact.

Fin regions, corresponding to morphotype III, and ventral regions, corresponding to morphotype IV, displayed more isotropic or reduced denticle forms accompanied by locally complex collagen networks. These findings are consistent with previous reports indicating that benthic and ventral surfaces may be associated with abrasion resistance and multidirectional load distribution rather than primarily hydrodynamic optimisation [1,3,5,6,10,11,22]. A useful ecological comparison is provided by the demersal shark *Scyliorhinus canicula*. Studies of its dermal denticles have considered their role not only in hydrodynamic interactions but also in surface protection and interactions with the external environment, including mechanisms potentially limiting biofouling [38]. In the examined specimen of *C. arabicum*, the reduced or more isotropic denticle forms observed in ventral and fin-associated regions, together with locally multidirectional collagen arrangements, may similarly be consistent with mechanical demands associated with substrate contact, abrasion, and tissue deformation. This interpretation remains hypothetical because abrasion resistance and regional mechanical properties were not directly tested.

Transitional regions, represented by morphotype V, showed pronounced local variability in both denticle morphology and collagen organisation, supporting the interpretation that integumentary regionalisation in *C. arabicum* forms a continuous structural gradient rather than a set of sharply separated regions.

Polarised light microscopy revealed that collagen architecture ranged from homoge-neous anisotropic systems to plywood-like, cross-lamellar arrangements. The presence of orthogonal and undulating patterns, together with periodicity confirmed by 2D FFT analysis, indicates that collagen organisation is highly ordered and regionally variable. These patterns may reflect local mechanical demands, although such functional interpretation remains inferential in the absence of direct mechanical testing [3,7,8,9,10,18,19]. The co-occurrence of specific denticle morphotypes with distinct collagen arrangements supports the interpretation of shark skin as a regionally differentiated composite tissue rather than as a uniform protective covering [1,2,3,4,5,6,10]. This is consistent with recent comparative studies showing that the mechanical behaviour of shark skin depends on species, body region, stress axis, ontogenetic stage, and ecomorphological context [5,6,7,8,9,10].

The close spatial association between dermal denticles and the collagen-rich dermis further suggests that denticles may participate not only in hydrodynamic modulation but also in mechanical load transfer across the skin. In the present material, denticle bases were embedded within collagenous dermal regions, indicating structural integration between mineralised surface elements and the fibrous connective tissue scaffold. This organisation may be particularly relevant in benthic species, in which the integument is exposed not only to water flow but also to repeated contact with the substrate.

Recent comparative studies of shark denticles further support the view that denticle morphology is highly diverse and functionally heterogeneous across shark lineages, with different morphogroups associated with variation in mechanical properties, protective capacity and hydrodynamic relevance [5,6]. In this context, the five morphotypes identified in *C. arabicum* should be interpreted as regional structural variants within a single species rather than as universal morphogroups. The reduced and more isotropic denticle forms observed on the ventral and transitional surfaces of the examined specimen appear more consistent with a benthic functional context involving surface protection, potential abrasion tolerance, and multidirectional loading [5,6,11,22] than with the strongly hydrodynamic specialisations commonly emphasised for pelagic sharks [4,12,13,14,15,16,17,36,37,38,39,40,41]. Comparisons with pelagic taxa remain useful for illustrating the broader functional diversity of shark skin, but they should be applied cautiously because swimming mode, frequency of substrate contact, and local mechanical demands differ substantially between pelagic and benthic species.

Accordingly, ecological and phylogenetic context should both be considered when interpreting regional integumentary specialisation in *C. arabicum*. Similar morphological features may have different or overlapping functional implications in pelagic and benthic sharks, and their presence alone does not demonstrate a specific biomechanical or hydrodynamic role.

The regional correspondence between denticle morphology and collagen arrangement should nevertheless be interpreted cautiously. Although comparable studies have shown that shark skin mechanics vary with body region, stress axis, ecomorphotype and developmental stage [7,8,9,10] the present investigation did not directly measure tensile properties, abrasion resistance or hydrodynamic performance. Therefore, the proposed biomechanical roles of the observed structures remain hypothesis-generating rather than experimentally demonstrated.

This study also has several limitations. The analysis was based on a single individual, and the two-dimensional nature of histological sections limits complete reconstruction of the three-dimensional collagen architecture. In addition, no mechanical assays, abrasion tests, or hydrodynamic modelling were performed. Therefore, the observed regional patterns should be interpreted as detailed morphological evidence of integumentary organisation in *C. arabicum*, not as population-level variation or direct functional proof. Future studies should combine comparative sampling across individuals and related benthic species with three-dimensional imaging, mechanical testing and flow-based analyses to verify the biomechanical implications proposed here. Inter-individual variability, sex-related differences, ontogenetic variation, and possible environmental effects on skin organisation could not be assessed and should be specifically addressed in future comparative studies.

## 5. Conclusions

The integument of *C. arabicum* represents a hierarchically organised, regionally differentiated composite system in which dermal denticle morphology and collagen fibre architecture show clear spatial correspondence. The identification of five dermal denticle morphotypes, together with region-specific collagen arrangements, indicates multi-level structural specialisation of the skin. The observed association between mineralised surface elements and the fibrous dermal matrix suggests that the integument of this benthic shark may be associated with regionally variable mechanical demands, including directional loading, substrate contact, and local tissue support. However, because the study was based on post-mortem material from a single individual and did not include mechanical or hydrodynamic testing, these functional implications should be treated as hypotheses requiring further verification. Overall, the study provides new morphological evidence for the integrated organisation and regional variability of elasmobranch skin.

These conclusions should be interpreted within the limitations of the present material, because the research describes one adult female and cannot resolve interindividual, sex-related, ontogenetic or environmentally induced variation.

## Figures and Tables

**Table 1:** Regional differentiation and characterisation of dermal denticle morphotypes in *Chiloscyllium arabicum*. These were distinguished based on diagnostic features of the crown and the degree of structural differentiation across the crown–neck–base axis. The spatial orientation of the denticle crowns exhibited regional variability, ranging from 22.33° to 53.09° based on regional mean values; however, these values were not directly assigned to specific morphotypes.

Morphotype	Body Regions	The Geometry of the Crown	Cusp	Surface Ornamentation	Differentiated Crown–Neck–Base	Arrangement of Denticles	Description of the Denticles
I (elongated ridge -bearing)	1, 2, 4, 6–10 (dorsal)	Elongated, anisotropic, laterally flattened	Present, directed caudally	Prominent longitudinal ridges (median and lateral) inter-ridge grooves, and surface micro-ornamentation	Well-defined	Imbricated	Pronounced anteroposterior asymmetry, with directional organisation of the crown
II (flattened tile-like)	5 (lateral)	Low-profile, isotropic, rhomboidal to sub-rectangular	None	Smooth or very subtle microtexture	Reduced (lacking a distinct neck)	Tessellated (mosaic-like) with edge-to-edge contact	Flattened crown, lacking directional structures
III (dome-shaped fin type)	11, 12, 13–15 (fins)	Low-profile to moderately domed, rounded, rounded-quadrangular	None	Smooth or very subtle microtexture	Limited; neck short or undifferentiated	Regular rows, parallel to the body axis	Domed crown; regional variability (more rounded vs. more regular)
IV (reduced plate-like)	3, 18–20 (ventral)	Very low-profile, low-domed, isotropic	None	Smooth, lacking ornamentation	Highly reduced (fused crown–base, neck absent)	Variable: ranging from densely packed to dispersed	Morphological reduction
V (transitional reduced)	16, 17 (transitional)	Low-profile, irregular and frequently asymmetrical	None	Smooth	Poorly defined	Irregular, with variable orientation	Intermediate form

**Table 2:** Organisation of collagen fibres in the skin regions of *Chiloscyllium arabicum*, determined based on birefringence analysis and fibre orientation. Cross-ply-oblique fibre arrangement; interwoven, intertwined network; orthogonal, perpendicular arrangement; plywood-like, multilayered structure with rotating fibre directions. Parameters are provided as representative values for dominant patterns. Functional interpretation is based on the observed collagen architecture and is inferential in nature.

Anatomical Region	Organisation Type	Main Features	Parameters (Selected)
Anterior to mid dorsal region	Anisotropic	Parallel bundles	~80.85° ± 8.26°; ~250 μm
Dorsal region beneath the denticles	Multi-layered cross-ply	Oblique interweaving	~45°; ~70 μm
Dorsal region	Periodic (2 populations)	Thick + thin fibres	~75/~15 μm; ~100/~12 μm
Transitional dorsal region	Periodic (mixed)	Interweaving scales	~80/~15 μm; ~45 μm
Caudodorsal region	Interwoven (multiscale)	Multiscale network	7–100 μm
Ventral region	Interwoven (intertwined)	Network + spaces	~15 μm; ~90 μm; ~40°
Nasal region	Periodic (crimped/undulated)	Fibre undulation	~−73.5°; 6–8 μm; ~140 μm
Fins regions	Orthogonal	Layers 0°/90°	~200 + ~200 μm
Deep trunk tissues	Plywood-like	Layered arrangement	~950 + ~1200 μm

## Data Availability

The data presented in this study are available on request from the corresponding author.
